# Toxicity and magnetometry evaluation of the uptake of core-shell maghemite-silica nanoparticles by neuroblastoma cells

**DOI:** 10.1098/rsos.231839

**Published:** 2024-06-19

**Authors:** Raúl López-Martín, Nieves Aranda-Sobrino, Nerea De Enciso-Campos, Elena H. Sánchez, Gregorio Castañeda-Peñalvo, Su Seong Lee, Chris Binns, Inmaculada Ballesteros-Yáñez, Jose A. De Toro, Carlos A. Castillo-Sarmiento

**Affiliations:** ^1^ Departamento de Física Aplicada, Instituto Regional de Investigación Científica Aplicada (IRICA), Universidad de Castilla-La Mancha, Ciudad Real 13071, Spain; ^2^ Department of Inorganic and Organic Chemistry and Biochemistry, School of Medicine, University of Castilla-La Mancha, Ciudad Real 13071, Spain; ^3^ Departamento de Química Analítica y Tecnología de los Alimentos, Facultad de Ciencias y Tecnología Química, Universidad de Castilla-La Mancha, Ciudad Real 13071, Spain; ^4^ NanoBio Lab, Institute of Materials Research and Engineering, 31 Biopolis Way, #09-01, The Nanos, Singapore 138669, Singapore; ^5^ BIomedicine Institute, Universidad de Castilla-La Mancha, Albacete 02008, Spain; ^6^ Department of Nursing, Physiotherapy and Occupational Therapy, School of Physiotherapy and Nursing, University of Castilla-La Mancha, Toledo 45071, Spain

**Keywords:** magnetometry, maghemite-silica, cellular uptake, nanoparticle toxicity

## Abstract

Nanoparticle uptake by cells is a key parameter in their performance in biomedical applications. However, the use of quantitative, non-destructive techniques to obtain the amount of nanoparticles internalized by cells is still uncommon. We have studied the cellular uptake and the toxicity of core-shell maghemite-silica magnetic nanoparticles (MNPs), with a core diameter of 9 nm and a shell thickness of 3 nm. The internalization of the nanoparticles by mouse neuroblastoma 2a cells was evaluated by sensitive and non-destructive Superconducting Quantum Interference Device (SQUID) magnetometry and corroborated by graphite furnace atomic absorption spectroscopy. We were thus able to study the toxicity of the nanoparticles for well-quantified MNP uptake in terms of nanoparticle density within the cell. No significant variation in cell viability or growth rate was detected for any tested exposure. Yet, an increase in both the amount of mitochondrial superoxide and in the lysosomal activity was detected for the highest concentration (100 μg ml^−1^) and incubation time (24 h), suggesting the onset of a disruption in ROS homeostasis, which may lead to an impairment in antioxidant responses. Our results validate SQUID magnetometry as a sensitive technique to quantify MNP uptake and demonstrate the non-toxic nature of these core-shell MNPs under our culture conditions.

## 1. Introduction

Magnetic nanoparticles (MNPs) are applied to an ever-broadening catalogue of theragnostic techniques [1], including magnetic hyperthermia therapy [2–6], field-guided therapies [7–9], biomarkers and cell/protein separation [10,11], contrast-enhanced magnetic resonance imaging (MRI) [12–16] or magnetic particle imaging [17–19]. Progress in these applications depends on biocompatibility assays, which consider the actual number of nanoparticles internalized by the cells. The accurate measurement of nanoparticle uptake is thus a crucial requirement for the validation of toxicology models, as well as for the investigation of optimum endocytosis in different cell types as a function of nanoparticle size, shape and architecture [20,21], an indispensable input for drug delivery and hyperthermia therapies [22]. Beyond rather qualitative microscopic techniques or semi-quantitative fluorescence approaches, the total nanoparticle uptake is conventionally quantified using different destructive mass spectroscopy techniques [20,23]. Another analysis technique that has been rarely used in cellular uptake measurements is graphite furnace atomic absorption spectroscopy (GFAAS) [24,25]. This technique allows the quantification of elements in small quantities of analyte with high sensitivity using an electric graphite-coated furnace to vapourize the sample. Unfortunately, GFAAS also calcinates any organic matrix that the analyte may contain, destroying the cells under study [26].

MNPs, however, enable the non-destructive evaluation of cellular uptake by high-sensitivity Superconducting Quantum Interference Device (SQUID) magnetometry and even facilitate monitoring of the nanoparticles fate through finer details of their magnetic behaviour using both static (DC) [27–31] and dynamic (AC) measurements [32–34]. Nevertheless, the relatively small amplitudes available in AC magnetometers (now commonly used to characterize the absorption rate for magnetic hyperthermia) are often unable to saturate the magnetic response and, thus, are unable to measure the mass of internalized MNPs [35]. Nonetheless, approaches to use AC biosusceptibility to quantify internalization have been made [36] although a need for a custom-made prototype discards them as a practical technique in research [36,37].

The second key point to ensure the applicability of the nanoparticles to medicine, as important as the characterization of the nanomaterials themselves (composition, size, morphology, etc., as reviewed in [1,38]), is the safe assessment of the nanoparticles' potential toxicity [39]. Since, for their application to *in vivo* therapies, MNPs must be biocompatible, an understanding of the interaction of the particles with living matter is indispensable for bench-to-bedside clinical studies [40]. In general, a lack of standardization in toxicology studies and uptake assessment still hampers the progress in developing risk-free MNPs, since comparison between experiments and reproducibility is not trivial [41]. Although superparamagnetic (SPM) Fe oxide nanoparticles (SPIONs) have been commercially available for MRI enhancement and hyperthermia therapy for some time [22], recent studies have raised a word of caution regarding their potential toxicity [42]. Therefore, coating with a thin silica (a well-known biocompatible material) shell appears convenient in order to increase the nanoparticles' safety, while also facilitating their functionalization with organo-silane molecules and fluorescent dyes [43]. The silica coating can be readily added by using Si-containing compounds such as tetraethyl orthosilicate (TEOS), usually in a second step after the synthesis of the MNPs [16,44].

In this work, we employ highly uniform 9 nm SPIONs (similar to those commercially available for enhanced MRI contrast) coated with a thin silica shell (approx. 3 nm in thickness). Mouse neuroblastoma 2 a cells (Neuro-2a) were exposed to a wide range of these particles for 24 h in order to test the magnetometry evaluation of the NP uptake against GFAAS spectroscopy and study its correlation with cell viability, cell growth and ROS homeostasis.

## 2. Results

### 2.1. Transmission electron microscopy characterization

**Figure 1.**
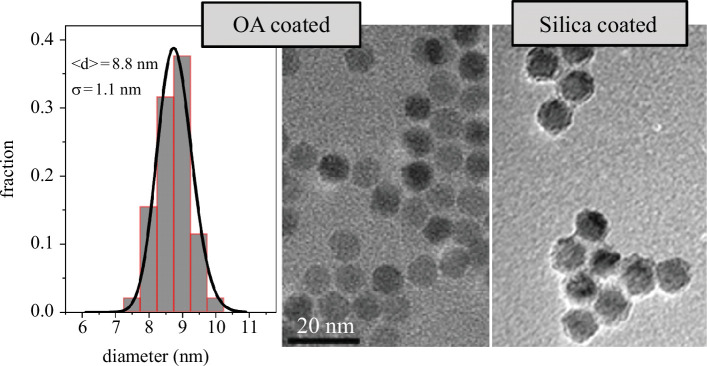
Transmission electron micrographs of oleic acid-coated (middle) and silica-coated (right) nanoparticles. The size distribution (left) was extracted from several images of oleic acid-coated particles and fitted to a lognormal function.

The maghemite nanoparticles used in this work were synthesized by thermal decomposition of iron pentacarbonyl (Fe(CO)_5_) with a subsequent oxidation stage that ensures the full oxidation of the nanoparticles by the addition of (CH_3_)_3_NO at high temperatures (more than 100°C) as described elsewhere [45]. Figure 1 shows transmission electron micrographs (TEM) of the oleic acid (OA)-coated MNPs (middle panel) and the silica-coated ones (right panel) prepared from them. The nanoparticles are spherical with a narrow size distribution (also shown in figure 1), which fitted to a lognormal distribution, yields an average size of 8.8 nm and a standard deviation of 1.1 nm. Thus, the polydispersity in our as-synthesized maghemite NPs is 12%, defined as the ratio of the standard deviation and the average size. When coated with a silica shell, the size of the nanoparticles increases, and the thickness of the shell is estimated to be around 3 nm. The silica coats the nanoparticles individually (rather than a cluster of them) thanks to the fine-tuned addition of TEOS in the solution of NPs, where Igepal was also added (see §4 and [44] for a detailed description).

**Figure 2.**
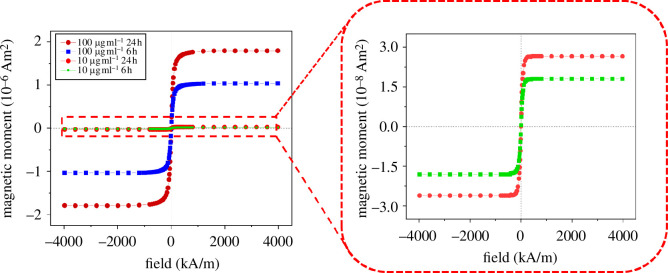
Magnetic response of cell cultures with particle concentrations of 100 and 10 µg ml^−1^ at 6 and 24 h of incubation time. Error bars are smaller than the datapoint size.

After the exposure of the cells to the maghemite NPs in different concentrations and for different exposure times, an extract was obtained by breaking cell integrity as described in §4. These extracts, containing the organelles and the internalized MNPs, are characterized by SQUID magnetometry. The magnetic response was measured at room temperature (300 K) upon the application of different magnetic fields, up to 4 MA m^−1^. Figure 2 shows the response after the proper diamagnetic background subtraction for cells exposed to 100 and 10 μg ml^−1^ solutions of silica-coated maghemite NPs for 6 and 24 h each. The corresponding negative control of the samples is shown in electronic supplementary material, figure S1. In figure 2*a*, typical SPM behaviour, that is, complete absence of loop hysteresis, is observed for all samples, as expected for 9 nm maghemite nanoparticles. The calculated blocking temperature, i.e. the temperature above which the MNPs show SPM behaviour, is around 50 K, far below 300 K (using magnetocrystalline anisotropy values of the order of 10^4^ J m^−3^ [46,47]). However, the maghemite@silica nanoparticles will be aggregated inside the cells, and interparticle interactions between MNPs will increase and so will the temperature at which the MNPs become SPM [28]. Nevertheless, even if this were the case, we expect this temperature to be below 300 K as shown by Andersson *et al*. for 9 nm bare maghemite NPs with strong dipolar interaction between them [47]. Moreover, the saturation magnetic moment (µ_s_) of the samples varies by two orders of magnitude: from 10^−6^ Am^2^ for the cells exposed to the most concentrated solution of NPs to 10^−8^ Am^2^ for cells exposed to the 10 μg ml^−1^ solution. Such a large change (faster than linear) in the absolute magnetic moment—and thus in the number of NPs within each cell—suggests that a threshold concentration is needed to start a significant internalization process. As expected, the lower the exposure time, the lower the nanoparticle uptake of the cells. On the other hand, focusing on the exposure time dependence (using the same solution of NPs), the magnetic moment (thus the internalization process) changes in a sublinear fashion. Although still far from it, this reflects the existence of saturation in the internalization process, as it has been reported, e.g. for polystyrene NPs [48]. Note that only two experiments were performed keeping one of the parameters fixed; therefore, a detailed assessment of the concentration and exposure time dependences is out of the scope of this work.

From the µ_s_ values, the iron content in the cells can be calculated. Note that µ_s_ is the absolute magnetic moment of the sample, thus directly proportional to the amount of magnetic material in each sample. As the number of cells in each sample is kept constant and the NPs are the only significant source of magnetic signal in the sample, dividing the value of µ_s_ by the saturation mass magnetization of maghemite directly gives the mass of NPs in the sample. Since the saturation magnetization of the NPs is not expected to change with their silica coating (no alloying whatsoever has been observed using our synthesis method [44,47]), the mass of the internalized nanomagnets has been determined using the saturation magnetization measured in the OA-coated nanoparticles in powder form (see electronic supplementary material, figure S2, for the M(H) response), namely 60.2 Am^2^ kg^−1^. The results of µ_s_ for all the samples are gathered in table 1. The uncertainty is ±1 over the last digit.

**Table 1.** Mass of iron internalized in the samples and in the negative controls (denoted by (**C**)) and corresponding average number of nanoparticles internalized by each cell, obtained from magnetic measurements.

sample	μ_**S**_ **(10** ^−**6**^ A m^**2**^ **)**	*m*_**Fe**_ **(**μg**)**	***m***_**Fe**_/cell (pg)	NPs/cell (**10** ^**5**^ )
100 μg ml^−1^ 24 h	1.790	20.80	27.8	211.9
100 μg ml^−1^ 24 h (**C**)	0.101	1.16	1.6	11.8
100 μg ml^−1^ 6 h	1.040	12.08	16.1	123.2
100 μg ml^−1^ 6 h (**C**)	0.068	0.81	1.1	8.3
10 μg ml^−1^ 24 h	0.026	0.30	0.4	3.1
10 μg ml^−1^ 6 h	0.018	0.21	0.3	2.1

From the maghemite mass and the volume of the core nanoparticles obtained by TEM (figure 1), the average number of MNPs within each cell, or NP uptake, can be calculated, since the number of cells in the samples is, on average, $7.5\times 10^{5}$ cells per sample. The value ranges from $2.12\times 10^{7}$ nanoparticles for cells exposed to the 100 μg ml^−1^ solution of MNPs for 24 h to $2.1\times 10^{5}$ nanoparticles, two orders of magnitude lower. These numbers are also presented in table 1. Note that, owing to factors such as the background subtraction, we have considered an uncertainty in the magnetic moment (10^−9^ Am^2^) at least one order of magnitude higher than the technical sensitivity of the SQUID magnetometer. Nevertheless, the sensitivity of this magnetometry technique is at least 10 ng, comparable to ICP-MS [20].

GFAAS, which consists of the quantification of a specific element by the absorption spectra of its atomic vapour [26], has also been performed to quantify the cellular uptake in our extracts and validate the less common magnetometry approach. To compare those values with the ones obtained by GFAAS, the internalized Fe mass, $m_{Fe}$ , is required. As the maghemite mass is known (as explained above) and so are the atomic masses of Fe and O, the values of $m_{Fe}$ and $m_{Fe}/cell$ (shown in table 1) can be calculated.

The results of both SQUID and GFAAS measurements are shown in table 2, along with the values of the control sample, i.e. samples with no cells where the protocol (see §4) was repeated in exactly the same way. As the sensitivity of GFAAS depends on the wavelength, those with the smallest amounts of Fe could not be quantified using a wavelength of 372 nm, with a detection limit of 50 ng ml^−1^. Instead, a wavelength of 248.3 nm, with a sensitivity down to 5 ng ml^−1^, was employed. The two wavelengths are complementary, for the latter one cannot quantify too high Fe contents (missing values in the right column in table 2).

**Table 2.** Mass of iron within the cells and in the control samples (denoted by (**C**)) quantified by magnetometry ( $m_{Fe,mag}$ ) and by GFAAS at different wavelengths.

	SQUID	GFAAS	
**s**ample	***m***_**Fe,mag**_ **(**µ**g)**	***m***_**Fe**_ **(**µ**g) @372 nm**	***m***_**Fe**_ **(**µ**g) @248.3 nm**
100 μg ml^−1^ 24 h	20.80	16.0 ± 1.3	-
100 μg ml^−1^ 24 h (**C**)	1.16	1.0 ± 1.5	0.88 ± 0.14
100 μg ml^−1^ 6 h	12.08	10.1 ± 1.1	-
100 μg ml^−1^ 6 h (**C**)	0.81	0.7 ± 1.5	1.25 ± 0.24
10 μg ml^−1^ 24 h	0.30	-	0.38 ± 0.16
10 μg ml^−1^ 24 h (**C**)	0*	-	0.15 ± 0.18
10 μg ml^−1^ 6 h	0.21	-	0.31 ± 0.17
10 μg ml^−1^ 6 h (**C**)	0*	-	0.08 ± 0.18

Interestingly, these values agree fairly well with the amount of Fe obtained by DC magnetometry. In the case of the sample with the highest amount of internalized MNPs, however, SQUID magnetometry overestimates the iron mass by 30%. Note that in table 2, the values of the control samples for the least concentrated solution of MNPs are 0 within the sensitivity of the SQUID magnetometer (thus, expressed as 0*). These values agree, within the error bars, with those obtained by GFAAS.

It is clear then, looking at table 2, that the main source of magnetic signal comes mainly from the MNPs within the cells, now in the extracts, and not from the remaining MNPs that have not been internalized: in terms of Fe mass, the values of the control samples do not constitute more than 7% of the total mass obtained by SQUID magnetometry in any case. Thus, the agreement between the values obtained by SQUID and GFAAS validates SQUID magnetometry as a probe of MNP uptake by cells.

The measured 27.8 pg Fe/cell for the cells exposed to the 100 μg ml^−1^ MNP solution (corresponding to *ca* 0.5 mM Fe) for 24 h is higher than the 3 pg Fe/cell found in the literature for THP1 cells using dextran-coated particles [28]. Interestingly, the concentration of these dextran-coated particles (in solution) is 40 times higher, i.e. 20 mM Fe. On the other hand, for Zn_0.5_Fe_2.5_O_4_@SiO_2_ core@shell MNPs exposed to human osteosarcoma MG-63 cells (200 μg ml^−1^, *ca* 0.8 mM Fe), the value obtained after 6 h of incubation was around 25 pg Fe/cell [49], somewhat higher than our measured Fe load (16.1 pg Fe/cell, see table 1) using half that NP concentration. Although comparison with the literature is difficult owing to the variety of incubation times, concentrations and sizes, these two examples point out the importance of the external shell owing to the electrostatic interactions with the plasma membrane [10].

### 2.2. Effect of MNP exposure on cell growth rate

**Figure 3.**
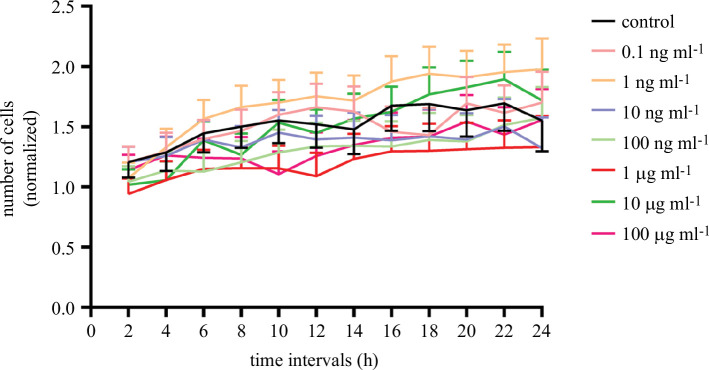
Effect of MNPs on Neuro-2a cell division. Neuro-2a cells were exposed to a wide range of MNP concentrations for 24 h. The effect of the exposure was measured by taking images of cell nuclei every 2 h. Cell quantification results were normalized for each experimental condition, taking as a reference the number of cells counted for each condition at the beginning of the experiment (*t* = 0). Histograms represent mean ± s.e.m. values.

**Figure 4.**
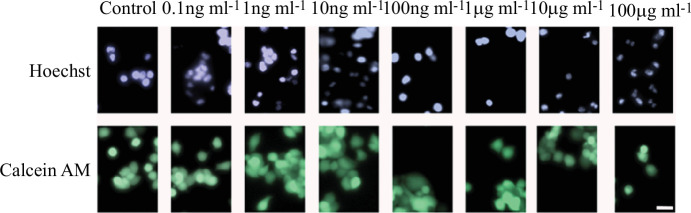
Representative fluorescence images of mouse Neuro-2a cells exposed to MNPs for 24 h. Neuro-2a cells were grown at different MNP concentrations for 24 h. Calcein-AM (green, lower panels) and Hoechst 33 342 (blue, upper panels) were used as cell-permeant dyes. Images were obtained using Cytation 5 cell imagining reader. Scale bar = 50 µm.

The effect of exposure to MNPs on the cell cycle of Neuro-2a cells was studied using Hoechst 33342, a fluorophore extensively used to stain cell nuclei, and a wide range of MNP concentrations. The effect of exposure to these particles was monitored by taking fluorescence images every 2 h for a period of 24 h. Under our experimental conditions, no significant differences in cell growth rate were observed at any of the seven concentrations studied (figure 3). Representative images taken by confocal microscopy are presented in figure 4.

### 2.3. Evaluation of cell viability

**Figure 5.**
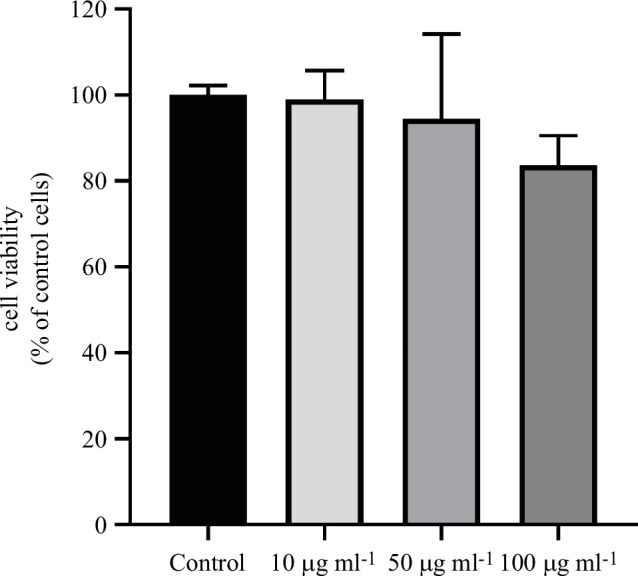
Effect of treatment with MNPs on cell viability on Neuro-2a cells. Effect on cell viability of Neuro-2a cells after treatment with different concentrations of MNPs (1, 50 and 100 µg ml^−1^) for 24 h. Cell viability was calculated as the ratio between the number of cells stained with Calcein-AM and the number of cells stained with Hoechst 33 342. The ratio under control conditions was taken as a reference. No significant differences were observed after treatment with different concentrations of MNPs on the viability of Neuro-2a cells. Data represented are mean ± s.e.m. of four independent experiments, each using different cell passages.

**Figure 6.**
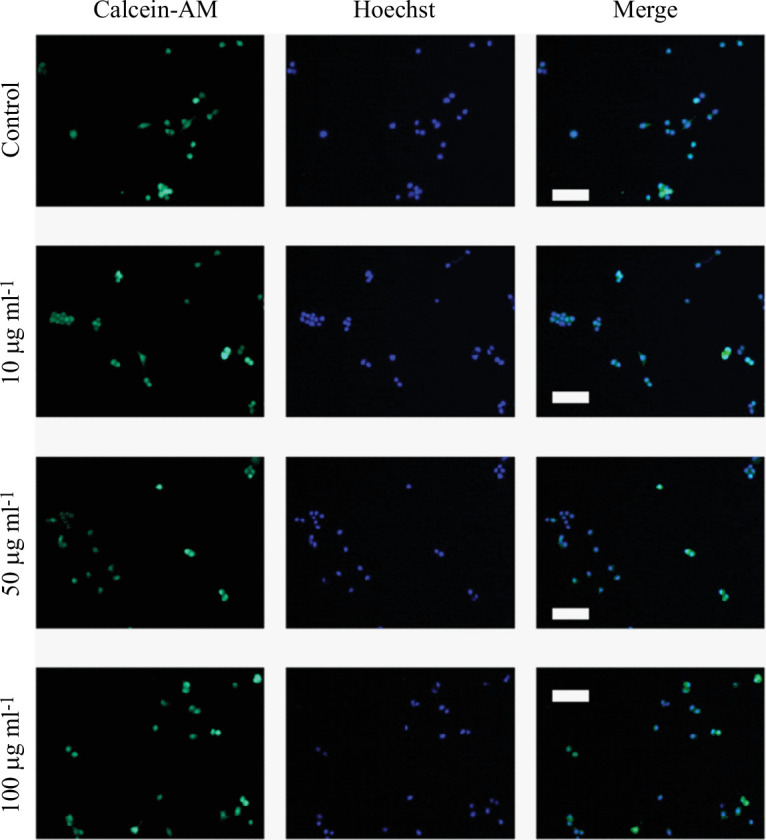
Representative fluorescence confocal images of the effect of treatment with MNPs on cell viability. Neuro-2a cells were exposed to MNPs (1, 50 or 100 µg ml^−1^) or maintained under control conditions for 24 h. For each experimental condition, representative images are shown presenting Calcein-AM staining (first column), Hoechst 33 342 staining (second column) and merge (third column). Scale bars represent 100 μm.

To study the effect of MNP exposure on cell viability, different fluorophores were used to differentiate between live and dead cells under different experimental conditions. Thus, three MNP concentrations (10, 50 and 100 µg ml^−1^) were selected, and Neuro-2a cells were subjected to 24 h MNP exposure periods or maintained in control conditions (figure 5). Under our experimental conditions, no significant changes in cell viability were observed as a consequence of cell exposure to MNPs. Representative images of these experiments are presented in figure 6.

Although the highest concentration used in this study, 100 μg ml^−1^, is usually one of the highest concentrations used in the description of the toxicological properties of a compound, it could be interesting, if a particular application requires it, to test the impact on cell viability of these particles at higher concentrations.

### 2.4. Evaluation of the intracellular effect of MNP exposure

**Figure 7.**
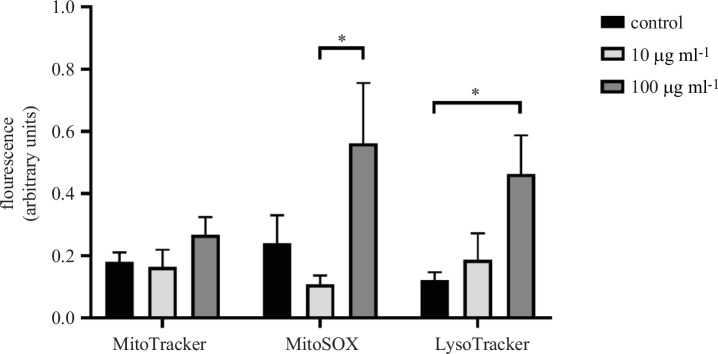
Intracellular effect of MNPs exposure on Neuro-2a cells. Neuro-2a cells were exposed to MNPs (10 or 100 µg ml^−1^) or maintained under control conditions, and the effect of the exposure on some intracellular organelles was measured. Three different fluorescent probes were used, MitoTracker, MitoSOX and LysoTracker, and the intensity of the fluorescence was measured after 24 h of MNPs exposition. Data represented are mean ± s.e.m. *p < 0.05 significantly different from the indicated condition according to the DMS post hoc test.

**Figure 8.**
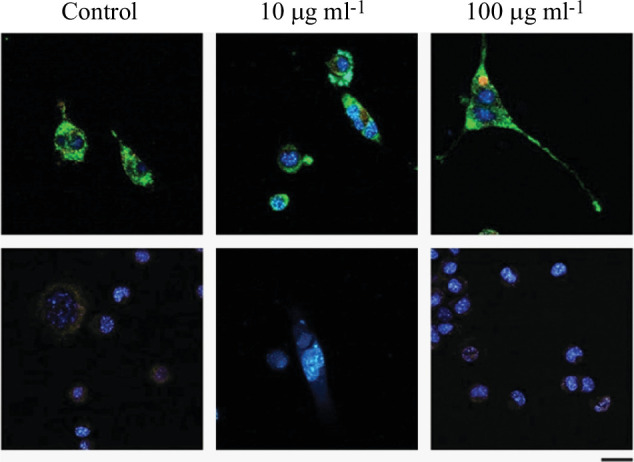
Representative fluorescence confocal images of the intracellular effect of MNP exposure. Neuro-2a cells were exposed to MNPs (10 or 100 µg ml^−1^) or maintained under control conditions, and the effect of the exposure on some intracellular organelles was measured. Columns show different experimental conditions, while rows show the type of staining performed: the first row shows MitoTracker in green, LysoTracker in red and Hoechst 33 342 in blue. The second row shows MitoSOX in red and Hoechst 33 342 staining in blue. Scale bar is 25 µm.

On completing the toxicological study of the effect of MNP exposure, we continued studying the intracellular variations that could take place as a consequence of the exposure to MNPs. To achieve this goal, we use three specific fluorescent probes: MitoTracker Green FM (MitoTracker), LysoTracker Red DND-99 (LysoTracker) and MitoSOX Red Mitochondrial Superoxide Indicator (MitoSOX). As no variations were reported in the cellular viability, we decided to use only two MNP concentrations in these experiments (10 and 100 µg ml^−1^). Therefore, Neuro-2a cells were exposed to a concentration of 10 or 100 µg ml^−1^ MNPs or maintained under control conditions for 24 h. After the incubation period, Neuro-2a cells were stained with different fluorescent probes and the intensity of the fluorescence was quantified (figure 7). In our experimental conditions, we did not observe variations in MitoTracker fluorescence intensity as a consequence of MNPs exposure but a significative (*p* < 0.05) increase in LysoTracker and MitoSOX fluorescence intensity was found when comparing cells exposed to a concentration of 100 µg ml^−1^ to control cells (280% of increase) or to cells exposed to 10 µg ml^−1^ (420% of increase), respectively. Representative images of these experiments are presented in figure 8.

Our results show that, under our experimental conditions, exposure of the Neuro-2a cell line to MNPs does not alter its viability. Although there are numerous studies that attempt to understand how MNPs interact with living matter, their conclusions are often inconsistent as these studies tend to show a high variability in terms of the type of particle used and the test conditions [50]. However, our results are consistent with those described by other authors in the literature with iron-containing particles in different *in vitro* models for exposures in the low μg ml^−1^ range for 24 h [51–53]. In addition, although the MTT method has been widely used to measure cell toxicity in cell cultures, through the variation of cell metabolic activity, in our case, the nature of our MNPs makes this method not viable, as they alter the spectrophotometric measurement especially in the μg ml^−1^ range (data not shown), as already described for other MNPs [53]. Real-time cell analysis, founded on impedance-based measurements, offers a compelling solution to circumvent interference concerns associated with nanoparticles, including those with silica coatings and fluorescence, and would be an accurate method to evaluate nanoparticle cytotoxicity in the future [54–57].

Although there is limited information about how core-shell maghemite-silica MNPs interact with living matter, fluorescent probes that facilitate the study of the impact of MNP exposure in certain subcellular organelles and their functionality have been used previously in other *in vitro* models [58–60]. Thus, we have used MitoTracker [61,62], which is not fluorescent in aqueous solution but becomes fluorescent in the mitochondrial environment and allows us to determine the amount of cellular mitochondria, MitoSOX [63], which penetrates into living cells and allows us to study mitochondrial superoxide production, and LysoTracker [64], which is retained in acidic compartments and allows us to quantify the amount of lysosomes. Our results show an increase in the amount of mitochondrial superoxide (134% increase at 100 µg ml^−1^ compared to control) while increasing lysosomal activity (278% increase at 100 µg ml^−1^ compared to control) as a consequence of cell exposure to MNPs. These results suggest that, under our experimental conditions, exposure to MNPs could disrupt ROS homeostasis and impair antioxidant responses. Furthermore, although the results of the experiments using LysoTracker should be taken with caution, given that lysosomes are responsible for the degradation of extracellular particles [65], it is possible that at the concentration of 100 µg ml^−1^ after 24 h of exposure, internalization of MNPs into cellular lysosomes would occur. In fact, it would not be unusual since all nanoparticles are processed through the formation of early-late endosomes and then lysosomes.

Taken together, as cell death has been reported to occur in proportion to the amount of ROS generated as a consequence of exposure to MNPs [66], these results suggest that the amount of MNPs and the exposure time used in these experiments are not sufficient to observe toxicity. These results agree with previous studies suggesting that the toxicity of nanoparticles depends on the concentration and exposure time of the cells, so that the longer the exposure time, the more damage would be observed as a consequence of increased cellular oxidative stress [67]. Therefore, it would be important when describing nanoparticles with clinical potential to demonstrate their effect at concentrations below the optimal concentration to avoid cell damage if treatment needs to be prolonged.

## 3. Conclusions

In summary, another proof of DC magnetometry as a tool to quantify the number of internalized MNPs in cells has been reported in this study. Both high and low concentrations of maghemite nanoparticles coated with silica (100 and 10 μg ml^−1^, respectively) have been quantified when neuroblastoma cells are exposed to them by measuring the saturation magnetic moment of the internalized particles. This indirect measurement of the iron mass within the cells agrees with GFAAS experiments, validating the method. Besides the magnetometry measurement of cellular uptake of these MNPs, their cellular viability has been studied, and, for the time window shown in this article, it can be concluded that these nanoparticles do not show any influence on cell growth or viability irrespective of the concentration used. However, it seems from fluorescence experiments that the intracellular environment is affected by MNPs when the concentration is 100 μg ml^−1^. Specifically, lysosome activity and oxidative stress increase, hinting at a disruption in ROS activity, which eventually could lead to cell death.

## 4. Material and methods

### 4.1. Nanoparticle synthesis

Maghemite nanoparticles (γ-Fe_2_O_3_) were synthesized by thermal decomposition of iron pentacarbonyl Fe(CO)_5_ in the presence of OA and dioctyl ether. A subsequent oxidation with (CH_3_)_3_NO is needed to complete the synthesis. The 9 nm maghemite nanoparticles were synthesized using the same experimental protocols as in [44]. Briefly, 10.4 mmol of OA and 30 ml of dioctyl ether were put in a three neck flask and heated up to 80°C overnight. Then, 4.56 mmol of Fe(CO)_5_ was added, and the solution was heated up to 100°C for 20 min after which it was further heated to 300°C in an Ar flow. At this temperature, the solution was refluxed for 90 min. The solution was then cooled down to 60°C, and 13.6 mmol of the oxidant agent [(CH_3_)_3_NO] was added. The solution was finally heated up to 120°C for 1 h and then heated slowly to 290°C where it remained for 1 h. After the resulting solution was cooled down to room temperature, acetone was used to precipitate the NPs, which were collected by centrifugation. A detailed description of the thermal decomposition synthesis can be found in [45].

After the synthesis of the maghemite nanoparticles, the solution of nanoparticles (around 10 mg) was added to an Igepal (6.8 mmol) and cyclohexane (16 ml) solution. Under stirring, 400 μl of an aqueous solution of 25 wt% NH_4_OH was also added. After stirring for 1 h, 100 μl of TEOS was added. After the TEOS addition, the microemulsion was stirred for 24 h, the silica shell thickness was checked by TEM and more TEOS was added if needed. Once the required thickness was obtained, methanol (5 ml) was used to break the microemulsion under stirring. After centrifugation, the silica-coated nanoparticles were separated and washed with ethanol. The resulting nanoparticles were dried in high vacuum conditions. A detailed description can be found in [68] and [44].

### 4.2. Reagents for the cell culture

Chemicals, culture media and culture plates used to obtain and maintain cellular cultures were acquired from Thermo Fischer Scientific (Waltham, MA, USA) unless otherwise stated. Products used in the fluorescence experiments were purchased from Sigma Aldrich (San Louis, MO, USA). All other products were of analytical grade.

### 4.3. Cell culture

Adherent mouse Neuro-2a cells were maintained under standard cell culture conditions. Briefly, Eagle’s minimum essential medium was supplemented with 10% fetal bovine serum (FBS), 1% antibiotics-antimycotics and 2 mM l-glutamine and cells were incubated in a humidified atmosphere supplied with 5% CO_2_ at 37°C.

Before experiments, cells were seeded in 6 well plates (at a density of 7.5 × 10^5^ cells/well), 24 well plates (10^5^ cells/well) or 96 well plates (2 × 10^4^ cells/well), as appropriate, and exposed to a range of MNP concentrations (from 0.1 to 100 μg ml^−1^) solved in distilled water or maintained under control conditions, during different periods of time.

### 4.4. Preparation of cellular extracts for Fe oxide quantification

Cells were plated in six well plates and exposed to 10 or 100 µg ml^−1^ MNPs for 6 or 24 h or maintained under control conditions. To measure the internalization of these particles by Neuro-2a cells, a parallel experiment was performed under the same conditions but without cells, referred to as ‘negative control’ in §2. At the end of the experiment, cells were washed twice with 1 ml of Hank’s balanced salt solution (HBSS), to eliminate unbound particles, and 100 µl of 1% sodium dodecyl sulfate (SDS) were added to each well to break down attached cells. The content of each well was transferred to an Eppendorf tube and evaporated using a speed vac (Eppendorf Concentrator Plus, obtained from Eppendorf, Hamburg, Germany) for 1 h at 60°C. Then, the amount of maghemite in the extracts was measured using two different methods, SQUID magnetometry and GFAAS, as described below.

### 4.5. SQUID magnetometry

An Evercool SQUID magnetometer from Quantum Design was used to register the magnetic response at room temperature (300 K) of the cell samples concentrated at the tip of the Eppendorf tubes described above, which were fitted to the usual SQUID straw holders. From the raw data obtained, the corresponding background subtraction was applied to all the samples.

### 4.6. GFAAS method

Atomic absorption measurements were made with a Varian absorption spectrometer model Spectra 400 equipped with a graphite furnace atomizer, an AS-50 autosampler, a Zeeman background correction system, pyrolytically coated plateau graphite tubes fitted with pyrolytic platforms and a hollow cathode iron lamp operated at 5 mA. The absorption was measured at 248.3 and 372.0 nm, with a slit width of 0.2 nm. In all cases, a 20 µl aliquot sample was injected into the graphite furnace by the autosampler. Argon was used as the inert gas, and each analysis was repeated at least three times to obtain the average value and its relative standard deviation.

The selected graphite atomizer temperature program is summarized as follows:

**Table:** 

step	temperature (°C)	ramp (s)	hold (s)	action
1	*95*	5	40	drying
2	120	10	0	drying
3	800	5	6	ashing
4	2300	1	2.1	atomization
5	2500	2	1	cleaning

Sample pretreatment acid digestion was performed with Parr Instrument Co. 4782 microwave acid digestion into PTFE bombs using aqua regia plus hydrofluoric acid. The system was heated at full power for over 90 s in a Samsung M6235 domestic microwave oven (800 W).

The concentration of iron was directly obtained using a calibration graph in ultrapure water (1% nitric acid).

### 4.7. MNPs effect on cell growth

Cells were plated into 96 well plates and loaded with cell-permeant dye Hoechst 33 342 (1 µM for 15 min) and exposed to a wide range of MNP concentrations (from 0.1 to 100 μg ml^−1^). Cytation 5 cell imagining reader (BioTek Instruments, Santa Clara, CA, USA), which allows us to maintain culture conditions (37°C and 5% CO_2_) for long periods of time and to systematically obtain images of each condition, was selected to host the experiment. Images were acquired automatically using a ×20 objective every 2 h taking two images for each condition until a complete treatment of 24 h was completed.

### 4.8. Cytotoxicity assays

Cells were plated into a 24 well plate and exposed to increasing concentrations of MNPs for 24 h (1, 50 or 100 µg ml^−1^) or maintained under control conditions. The effect of the MNPs on cell viability was measured using different fluorophores. Briefly, Calcein-AM was used as a cell-permeant dye to stain live cells (green fluorescence), and Hoechst 33 342 was used as a permeant dye to stain all cell nuclei (blue fluorescence). Therefore, cell viability could be calculated as the ratio between the number of green cells (live cells) and the number of blue cells (all the cells). At the end of the period of exposure to MNPs, cells were washed twice with HBSS, to discard unbound particles and incubated for 15 min with Calcein-AM and Hoechst 33 342 (1 µM, each). After this incubation, cells were washed twice with HBSS and maintained in complete medium during image acquisition by confocal microscopy, as described below.

### 4.9. Cell quantification

Cell counting was done using ImageJ [69] by blind data collection, using the Cell Counter plugin in order to automate the process, obtaining for each image the number of fluorescent cells (for each colour).

### 4.10. Intracellular impact of MNP exposure

Cells were plated into a 24 well plate and exposed to increasing concentrations of MNPs for 24 h (10 or 100 µg ml^−1^) or maintained under control conditions. MitoTracker, MitoSOX and LysoTracker were selected as probes capable of staining live cell compartments. After MNP exposition, staining was performed following manufacturer instructions and confocal images were captured of each condition.

### 4.11. Confocal microscopy and fluorescence measurements

Images per experimental condition were taken with a Zeiss LSM 800 (Carl Zeiss, Weimar, Germany) confocal microscopy system. For each experiment, two representative images were taken per cell well.

Fluorescent images for each condition in the viability experiment were taken as duplicates and acquired using a Plan Apochromat 10x/0.45 objective with one excitation laser at 494 nm for Calcein-AM and another laser at 348/455 (blue excitation/emission values) for Hoechst 33 342.

For the measurements of fluorescence intensity, images were obtained using a Plan Apochromat 20x/0.8 objective under the same acquisition conditions. The intensity of the fluorescence was measured using Fiji [70], with correcting values obtained using the number of cells in each image. MitoTracker was captured with one excitation laser at 490/512 nm (green excitation/emission values), MitoSOX with one excitation laser at 510/580 nm (red excitation/emission values) and LysoTracker with one excitation laser at 578/589 nm (red excitation/emission values). All captures were made by different channels in the confocal microscope.

### 4.12. Statistical data analysis

The statistical software SPSS, v. 24 for Windows (IBM Corp., Armonk, NY, USA) was used to examine differences between treatments. Statistical analysis was performed using a one-way ANOVA, followed by DMS post hoc test. Differences between mean values were considered statistically significant at *p* < 0.05 (95% confidence interval). To summarize the data in graphical representation, the software selected was the GraphPad Prism 8 program for Windows (GraphPad Software, San Diego, CA, USA).

Unless stated otherwise, a minimum of two experiments were performed using different cell passages; each experiment was performed in duplicate.

## Data Availability

All the data in this work can be accessed at the following Dryad Digital Repository [71]. Electronic supplementary material is available online at [72].
